# Mask-wearing behavior patterns among dental patients in post-pandemic China: a cross-sectional study

**DOI:** 10.3389/fpubh.2025.1617357

**Published:** 2025-08-11

**Authors:** Junfei Zhu, Fang Lin, Xuguang Yuan

**Affiliations:** ^1^Stomatology Center, China Japan Friendship Hospital, Beijing, China; ^2^Department of Pulmonary and Critical Care Medicine, China Japan Friendship Hospital, Beijing, China

**Keywords:** mask-wearing, post-pandemic, cross-sectional study, infectious disease, aerosol

## Abstract

**Background:**

The present study investigated the demographic characteristics of individuals who wear masks when visiting the Dental Department of a public Hospital in Beijing, China, 2 years after the Chinese government relaxed mask mandates following the COVID-19 pandemic.

**Methods:**

A cross-sectional study was conducted at the Dental Department of China–Japan Friendship Hospital. Patients visiting the Clinic of the Stomatology Center were observed. Data on mask-wearing status, age, gender, outdoor temperature, and weather conditions were recorded. Statistical analyses included chi-squared tests and binary logistic regression to identify predictors of mask use.

**Results:**

Among 1,009 patients, 46.3% wore masks. Females exhibited significantly higher mask-wearing rates than males (62.0% vs. 19.6%, OR = 6.457, *P* < 0.001). The increase of age positively influenced the mask-wearing rates (OR 1.010, *P* = 0.046). Lower temperatures (0–19°C) correlated with higher mask wearing rates (56.2–57.0%) versus warmer groups (20–39°C, 38.6–42.0%, *P* < 0.001), with each degree increase reducing likelihood by 2.3% (OR = 0.977; *P* = 0.007). Significantly elevated mask-wearing rates were observed during foggy weather conditions.

**Conclusion:**

Nearly half of patients continue to wear masks when visiting the Dental Department in the post-pandemic era. The mask wearing behavior was influenced by gender, age, and environmental factors such as outdoor temperature and weather. Females and older individuals showed higher adherence, while colder temperatures and foggy weather correlated with increased mask use.

## Introduction

Started from the COVID-19 pandemic, wearing mask became one of the most visible and widely adopted strategies among the numerous preventive measures implemented to mitigate the spread of the virus (1). Governments worldwide instituted mandates requiring the public to wear masks in the early stages of the pandemic, especially in indoor spaces and crowded environments (2). Consistent mask use reduced infection rates, and contracting SARS-CoV-2 may subsequently alter masking behavior in high-risk settings (3). Over time, as the global situation improved and the pandemic was brought under control, mask mandates were relaxed. However, the behavior of mask-wearing, once a public health necessity, continues to be an important indicator of individual and collective health awareness, even in the post-pandemic era (4).

The treatment procedures in dental Clinics often involve close contact with patients, aerosol-generation, and prolonged mouth opening. Additionally, splatters occurring during oral surgery procedures, could be also contaminated by respiratory pathogens, which make these environments potential hotspots for infectious disease transmission (5, 6). During the pandemic, dentists globally faced significant anxiety and fear. Many dental departments modified services to provide only emergency care per guidelines or closed indefinitely (7, 8). Notably, even as COVID-19 incidence declines, persistent transmission risks for other airborne pathogens including influenza viruses and *Mycoplasma pneumoniae* still persist in public environments (9). Given the unique characteristics of dental Clinics and the evolving nature of post-pandemic public health behaviors, it is of significance to explore how mask-wearing habits have persisted, or declined, within these specialized clinical environments.

Previous research has examined various factors that influence dental clinic visiting as well as mask-wearing behaviors, including gender, age, education level, smoking habits, and occupation (10–12). So far, few recent literature have examined the mask-wearing status in post-COVID-19 era. The survey reported by Lertsakulbunlue et al. (13) highlighted that the sustaining mask-wearing behavior in rural older adult populations in Thailand was significantly mediated by motivation and social norms. Additionally, post-COVID studies also suggested that psychological factors such as self-perceived facial attractiveness and social anxiety influenced the willingness to wear a mask (14, 15).

The present study aimed to evaluate current mask-wearing behavior and analyze influencing sociodemographic and environmental factors—such as age, gender, weather conditions, and outdoor temperature—among patients visiting the Dental Department of a public Grade A hospital in Beijing, China. The study was conducted 2 years after the relaxation of mandatory COVID-19 restrictions. This research would contribute to the understanding of how public health interventions, such as mask-wearing, are influenced by socio-demographic factors, and will provide valuable insights for healthcare providers and policymakers in shaping ongoing infection control strategies.

## Methods

The present study was conducted at China-Japan Friendship Hospital, Chaoyang District, Beijing, China. Patients visiting the Maxillofacial Clinic of the Stomatology Center during July 1st, 2024 to December 1st, 2024 were observed by one independent doctor (Junfei Zhu). The mask-wearing status of the patients was observed and recorded at the time they stepped into the Clinic room. The mask-wearing status, age, and gender were recorded. Additionally, outdoor temperature and weather conditions were documented using data from the mobile application *Moji Weather*. The inclusion criteria comprised patients who have scheduled an appointment for the day and presented for treatment. The persons who accompanied the patients to the Clinic were not included.

The scope of the Maxillofacial Clinic primarily focuses on tooth extraction, including impacted wisdom teeth, residual teeth, and loose teeth, as well as minor surgeries involve small cysts, tumors, abscesses, and wounds.

A preliminary investigation was conducted prior to the present study, in which 100 patients were observed. Among them, 48 patients wore masks, while 52 did not. Based on this data, the estimated mask-wearing rate was determined to be 48% (*P* = 0.48).

Using this estimate and assuming a margin of error (*δ*) of 0.05 and a standard normal critical value (*Z α*/2) of 1.96, following formula was applied:

$n=\frac{Z_{\alpha /2}^{2}P(1-P)}{\delta^{2}}$

Substituting the given values, the minimum required sample size was calculated to be *n* = 384.

The statistical analysis was performed using SPSS version 27.0. The Chi-squared test was employed to compare mask-wearing rates across different categories, including gender, age, underlying health conditions, weather, and outdoor temperatures, a *P* value <0.05 was considered statistically significant, and a *P* value <0.01 was considered highly significant. Binary logistic regression was used to analyze the influence of different included categories on mask-wearing behavior. The results of the regression analysis are presented as odds ratios (ORs) with 95% confidence intervals (CIs). Also, a *P* value <0.05 was considered to be statistically significant.

## Results

The present study spanned 184 days. A total of 1,009 patients were observed in the present study, including 629 females and 380 males, with ages ranging from 5 to 90 years. Among them, 865 were healthy, while 144 had systemic disorders, including cardiovascular and cerebrovascular diseases (*n* = 75), diabetes mellitus (*n* = 26), a history of allergies (*n* = 14), and other disorders (*n* = 28). The highest recorded outdoor temperature was 36°C, and the lowest was 3°C. During the study period, 500 patients visited the clinic on cloudy days, 323 on sunny days, 112 on foggy days, and 74 on rainy days (Table 1).

**Table 1:** Total characteristics.

Gender		Age		Systemtic condition		Temperature		Weather		Diagnoses	
Male	380	<15	21	Healthy	865	0–10 °C	163	Cloudy	500	Wisdom tooh	638
Female	629	15–34	619	Systematic disorders	144	11–20 °C	218	Foggy	112	TMD	152
		35–64	289			21–30 °C	412	Rainy	74	Other tooth need to be extrcted	122
		>64	154			31–40 °C	216	Sunny	323	Mass or tumor	52
										Oter diagnoses	43

**Figure 1:**
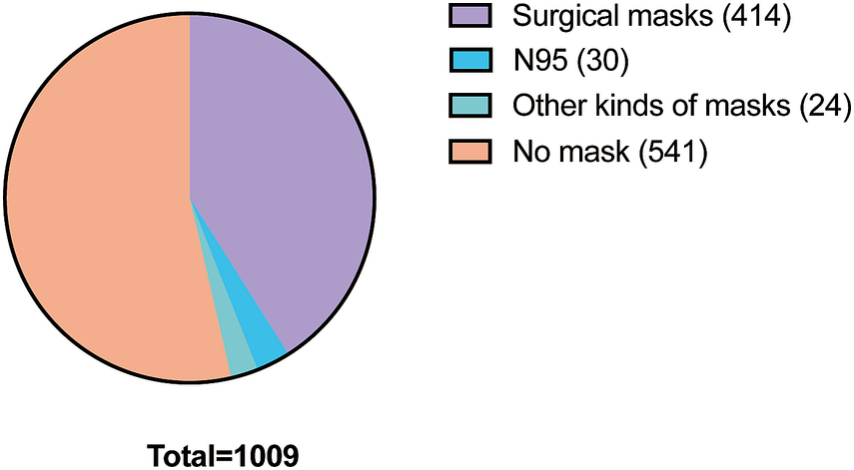
The overall mask-wearing status of the patients.

Of the 1,009 patients, 541 did not wear a mask, while 468 wore a mask. The overall mask-wearing rate was 46.3%. Most of the patients who wore masks chose surgical masks (*n* = 414), 30 patients wore N95 masks, and 24 patients wore other kinds of masks such as cloth masks, anti-dust masks and UV protection masks (Figure 1).

**Figure 2:**
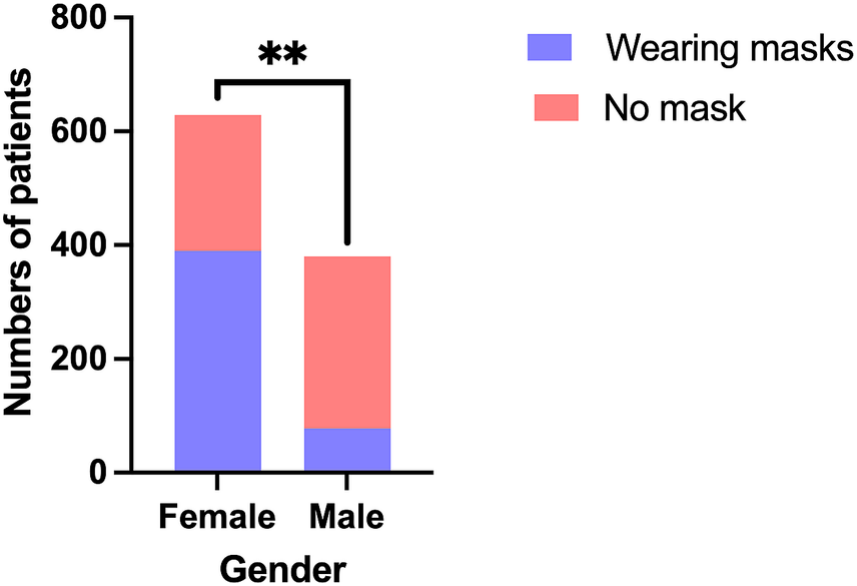
The comparison of mask-wearing rates between genders. **P < 0.001.

The mask-wearing rate among females was significantly higher than that of males (62.0% vs. 19.8%; *χ*^2^ = 163.868; *P* < 0.001) (Figure 2).

**Figure 3:**
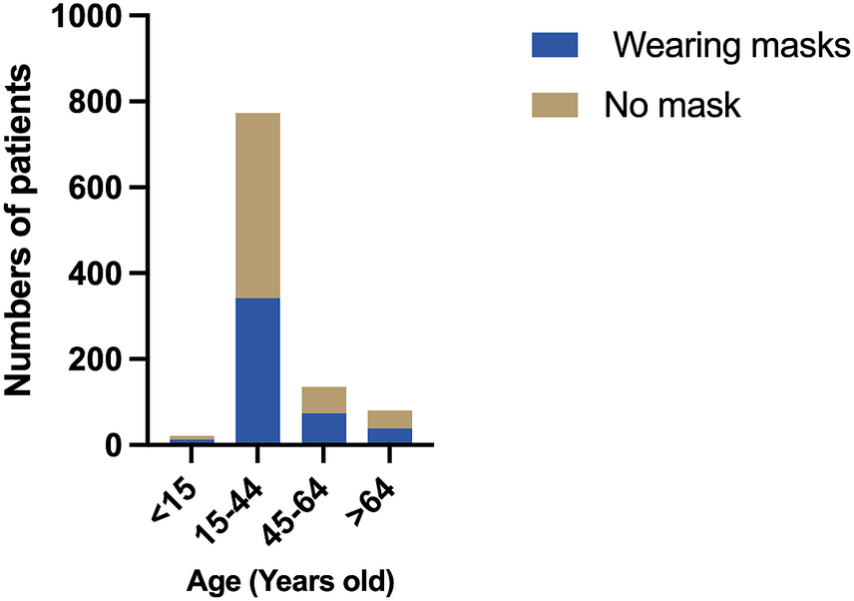
The comparison of mask-wearing rates between different age groups.

Age groups were categorized based on the guidelines provided in *Basic Epidemiology*, a book published by the World Health Organization (WHO), which recommends four age categories: 0–14, 15–44, 45–64, and ≥65 years (16). The 0–14 age group had the highest mask-wearing rate (61.9%), while the 15–44 age group had the lowest rate (44.2%). However, no statistically significant difference in mask-wearing rates was observed between the groups (*χ*^2^ = 7.497; *P* = 0.058) (Figure 3).

**Figure 4:**
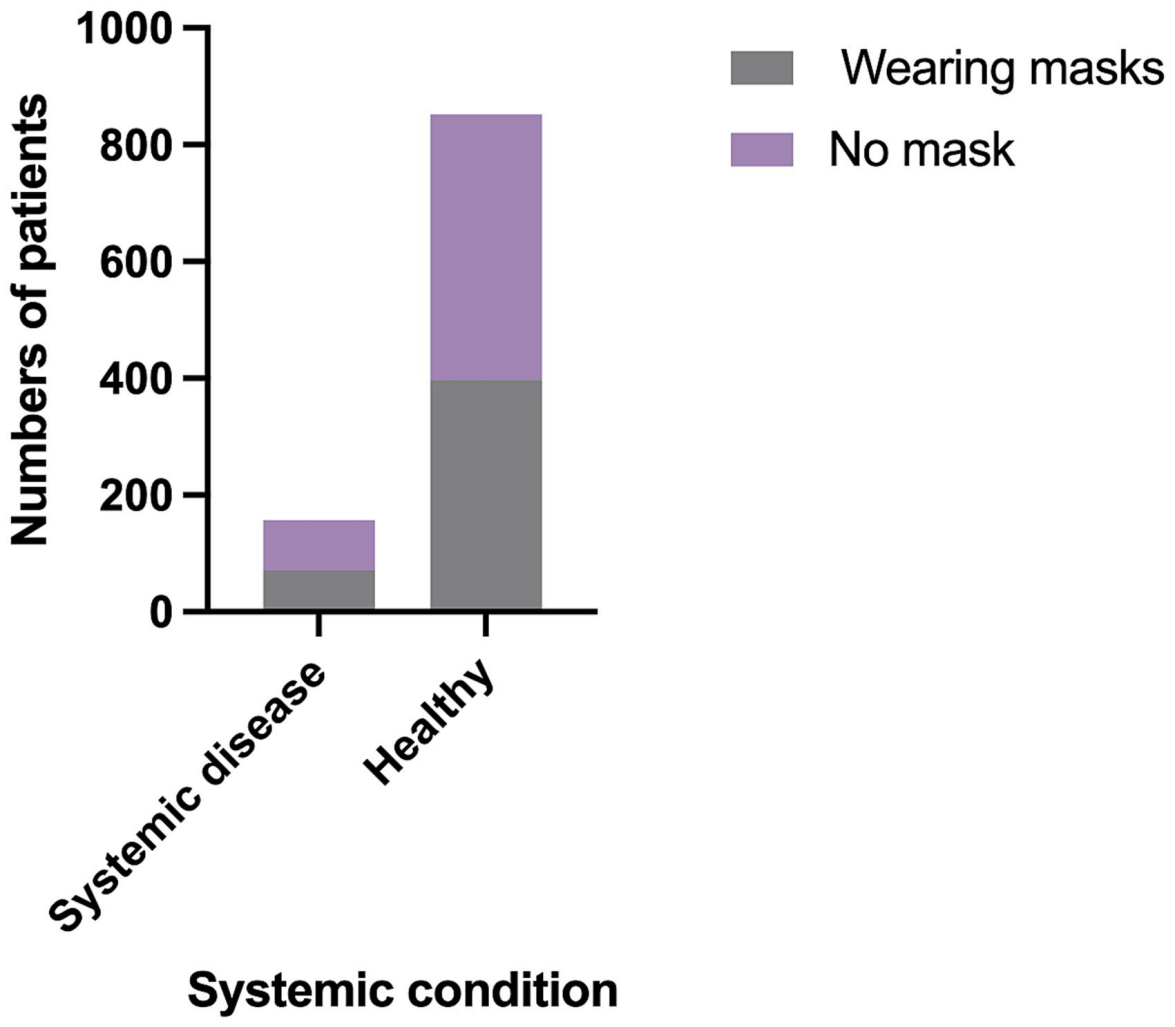
The comparison of mask-wearing rates between healthy patients and patients who have systemic diseases.

Patients with any systemic disorders, including cardiovascular and cerebrovascular diseases, diabetes mellitus, a history of allergies, and other conditions, were grouped into one category. The mask-wearing rate among patients without systemic disorders was 46.4%, while the rate among those with systemic disorders was 46.5%. No statistically significant difference was found between the two groups (*χ*^2^ = 0.001; *P* = 0.97) (Figure 4).

**Figure 5:**
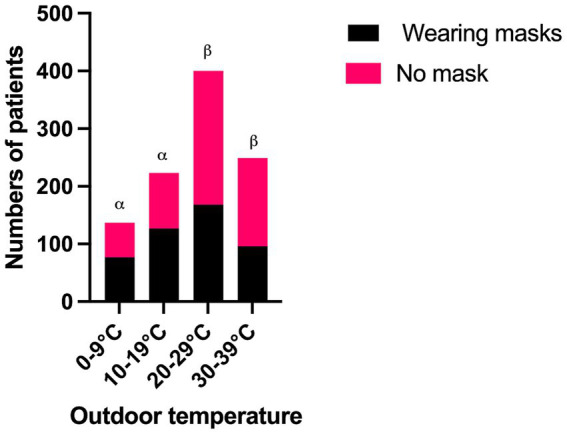
The comparison of mask-wearing rates between different groups of outdoor temperature. *α*, *β*: *P* < 0.05 between the groups of *α* and *β*.

The outdoor temperature was divided into four 10°C groups: 0–9°C, 10–19°C, 20–29°C, and 30–39°C. The mask-wearing rates among patients in these groups were 56.2, 57.0, 42.0, and 38.6%, respectively. Notably, the two lower-temperature groups (0–9°C and 10–19°C) had significantly higher mask-wearing rates compared to the warmer groups (20–29°C and 30–39°C) (*χ*^2^ = 24.554; *P* < 0.001) (Figure 5).

**Figure 6:**
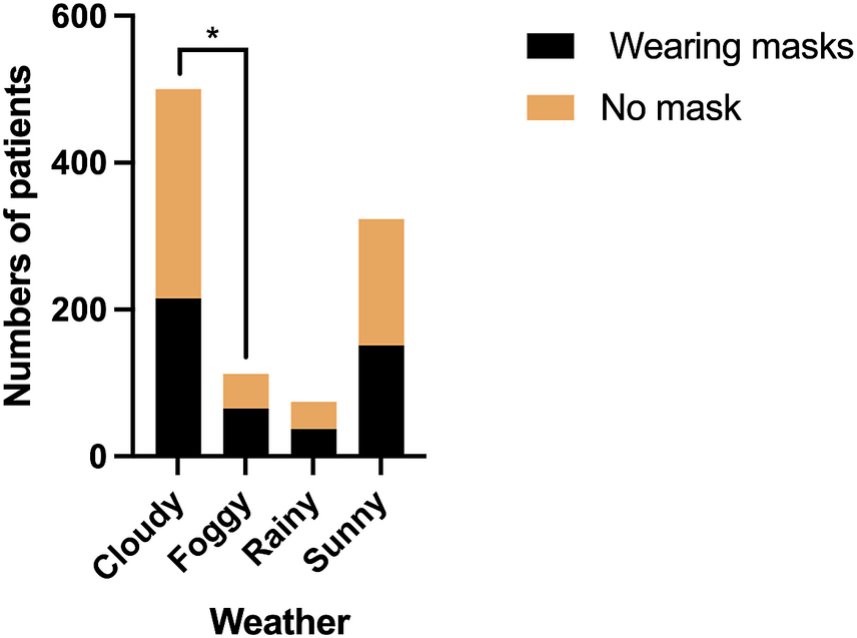
The comparison of mask-wearing rates between different groups of weather. *P < 0.05.

The mask-wearing rates of patients in different weather conditions were as follows: 43.0% on cloudy days, 58.0% on foggy days, 50.0% on rainy days, and 46.7% on sunny days. The mask-wearing rate on foggy days was significantly higher than on cloudy days (*χ*^2^ = 8.823; *P* = 0.032) (Figure 6).

The result of binary logistic regression showed females compared with male were more likely to wear masks (OR 6.457; 95%CI 4.769–8.744; *P* < 0.001); the increase of age positively influenced the mask-wearing rates (OR 1.010; 95%CI 1.000–1.020; *P* = 0.046); The increase of outdoor temperature negatively influenced the mask-wearing rates (OR 0.977 95%CI 0.961–0.994; *P* = 0.007) (Table 2).

**Table 2:** The results of binary logistic regression.

OR (95% CI)		*P*
Gender		
Male	1.000	
Female	6.457 (4.769–8.744)	<0.001**
Age	1.010 (1.000–1.020)	0.046*
Systemic disease	0.971 (0.624–1.512)	0.897
Temperature	0.977 (0.961–0.994)	0.007**
Weather		
Sunny	1.000	
Cloudy	0.930 (0.677–1.278)	0.656
Foggy	1.208 (0.735–1.988)	0.456
Rainy	1.210 (0.693–2.112)	0.503

*P < 0.05; **P < 0.01.

## Discussion

During the COVID-19 pandemic, extensive research was conducted on mask-wearing behaviors, examining various influencing factors such as age, gender, educational background, and other demographic characteristics (1, 17, 18). However, studies exploring mask-wearing habits in the post-pandemic era remain limited. Recent research has begun to address this gap. For instance, Lertsakulbunlue et al. (13) investigated mask-wearing behaviors among older adult individuals in rural Thailand through surveys and qualitative interviews. Their findings highlight that motivation and social norms are key determinants of continued mask usage in the post-COVID period. Additionally, Cha et al. (15) examined the influence of self-perceived facial attractiveness on mask-wearing intentions. The study suggests that, while masks primarily served as a protective measure during the pandemic, they have increasingly become a tool for self-presentation in the post-pandemic era. These studies provide valuable insights into the evolving psychological and social drivers of mask usage beyond health concerns.

The present study is the first to explore post-COVID mask-wearing behaviors in a first-tier city in China. China-Japan Friendship Hospital is a grade A class 3 Hospital in China. The current Hospital policy for mask-wearing includes recommending that all patients wear masks while in the Hospital, and mandating that all dental healthcare workers wear masks, protective suits and protective face masks throughout their work process. Based on preliminary investigation and previous studies (10, 19), he hypotheses of this study were that nearly half of the observed population would wear masks, and that the mask-wearing rate would be influenced by gender, age, outdoor temperature, and weather. The results are consistent with these hypotheses.

Masks in dental settings are essential to block harmful aerosols (like saliva, blood, and germs) released during procedures such as drilling or cleaning. They protect both staff and patients by preventing cross-contamination—stopping germs from passing between mouths, noses, or eyes (20). As observed, 46.3% of dental patients continued to wear masks in the post-pandemic era, suggesting that while the majority had stopped, a significant proportion still adhered to mask-wearing practices, especially in the high risk area like a Dental Clinic. This aligns with previous studies showing that mask-wearing remains important even after mandates were lifted due to lingering concerns about airborne disease transmission (21). Additionally, past research has emphasized that the persistence of such behaviors is influenced by both individual health concerns and societal norms (22). A study in Hong Kong used an online survey to explore why people kept wearing masks after the removal of the mandatory mask. The results showed that personal attitudes toward masks were the strongest predictor, followed by subjective norms and perceived self-efficacy (23). The analysis reported by Kawase et al. (24) suggested that emphasizing self-protection as a motivation for mask-wearing remained a consistently effective strategy throughout the pandemic, even as its duration extended. Promoting masks successfully encouraged sustained behavioral change among individuals over time.

The observed gender difference aligns with previous researches suggesting that women are generally more health-conscious and more likely to engage in preventive behaviors, including mask-wearing (19, 25). Psychological and sociocultural factors may also contribute to this disparity (26). Women may perceive greater personal or societal expectations regarding health protection and are often more engaged in caring for others, further reinforcing their likelihood to wear masks (27).

The binary logistic regression analysis demonstrated that advancing age was positively associated with higher mask-wearing rates. However, the Chi-squared test comparing age groups revealed a marginally non-significant result (*P* = 0.058), which could be explained by the limited sample size and the method of age-group categorization. The higher mask-wearing rate among younger children could be attributed to parental influence, as caregivers may ensure that children wear masks in public environments (28). As reported by Zhang et al. (29) during the pandemic, the strategy of sheltering combined with mask-wearing for individuals aged 50–64 years old was most effective, decreasing attack rate, Hospitalizations, and deaths by over 82%, indicating the importance of mask-wearing behaviors in aged population. In the present study a slight increase in mask-wearing rate with older age groups could be observed, which may be due to heightened health concerns and vulnerability to respiratory diseases, especially among older adult individuals who are at higher risk of severe illness (30).

The inverse relationship between outdoor temperature and mask-wearing rates is a notable finding. Patients in colder temperature groups (0–9°C and 10–19°C) exhibited significantly higher mask-wearing rates (56.2 and 57.0%, respectively) compared to those in warmer groups (42.0% at 20–29°C and 38.6% at 30–39°C; *P* < 0.001). Binary logistic regression further corroborated this trend, showing that each degree increase in temperature reduced the likelihood of mask-wearing by 2.3% (OR = 0.977; *P* = 0.007). This aligns with studies suggesting that thermal discomfort in warmer climates discourages prolonged mask use due to perceived breathability challenges and physical irritation (31). Conversely, colder temperatures may enhance mask acceptance, as masks can provide incidental benefits such as protecting asthmatics against the effects of exercise and cold air (32).

However, the sharp decline in mask use above 20°C suggests that temperature thresholds influence behavior. This could reflect cultural or regional norms in Beijing, where winter air quality concerns (e.g., particulate pollution) and seasonal influenza may compound health motivations for mask use, whereas summer heat dominates comfort priorities (33, 34).

Weather conditions also influenced mask-wearing behavior. The elevated mask use during foggy weather likely stems from associations between fog and air pollution in urban China, where residents often link reduced visibility to poor air quality and respiratory risks (35). Basic preventive strategies, such as dietary supplementation of vitamins, and mask wearing outdoors seem to be sufficient to minimizing the adverse impact of daily exposure to fog and haze (36). The pre-pandemic studies also showed that air quality alerts independently drive mask-wearing, even absent infectious disease threats (37).

While this study provides valuable insights into post-pandemic mask-wearing behaviors, several limitations should be acknowledged. First, the study’s single-site design in Beijing limits generalizability to rural areas or regions with differing climatic or cultural norms. Secondly, self-reported health conditions and systemic disorders were not verified through medical records, potentially introducing misclassification bias. Further, the analysis did not account for air quality indices (e.g., PM2.5 levels), which may confound the observed relationships between the present included factors and mask-wearing. Additionally, daily temperature averages might conceal intraday fluctuations affecting real-time masking decisions. Future studies should integrate real-time environmental monitoring and multi-site sampling to address these gaps.

In conclusion, the present study highlights the persistence of mask-wearing behavior among dental patients in post-pandemic Beijing, with nearly half continuing to use masks despite relaxed mandates. Gender emerged as the strongest predictor, with women three times more likely to wear masks than men. Age, and environmental factors—notably colder temperatures and foggy weather—also significantly influenced the adherence of mask wearing. This study offers several clinical implications for dental institutions and policymakers: First, mask dispensers at clinic entrances could be maintained year-round, especially targeting male patients. Second, patient reminders should be seasonally adjusted—emphasize mask benefits more proactively in warmer months when compliance drops sharply. Moreover, public health campaigns linking masks to broader protective benefits (e.g., against pollution or fog) should be developed to sustain compliance with mask-wearing beyond the pandemic context. Future multi-center studies across diverse populations and settings are warranted to validate these findings and track longitudinal trends in mask-wearing behavior. Additionally, different perspectives, such as the psychological and behavioral drivers for mask-wearing willingness are also worthy to be explored.

## Data Availability

The original contributions presented in the study are included in the article/supplementary material, further inquiries can be directed to the corresponding authors.

## References

[1] Sugimura M Chimed-Ochir O Yumiya Y Ohge H Shime N Sakaguchi T. The association between wearing a mask and COVID-19. Int J Environ Res Public Health. (2021) 18:9131. doi: 10.3390/ijerph18179131, PMID: 34501719 PMC8431493

[2] Scott N Saul A Spelman T Stoove M Pedrana A Saeri A. The introduction of a mandatory mask policy was associated with significantly reduced COVID-19 cases in a major metropolitan city. PLoS One. (2021) 16:e0253510. doi: 10.1371/journal.pone.0253510, PMID: 34288910 PMC8294480

[3] Pham D Lomeli A Goldhaber NH Valentine HD Knight R Longhurst CA. Longitudinal assessment of the impact of COVID-19 infection on mask-wearing behaviors. BMC Public Health. (2024) 24:2230. doi: 10.1186/s12889-024-19776-0, PMID: 39152377 PMC11328381

[4] Li J Yin J Ramakrishna S Ji D. Smart mask as wearable for post-pandemic personal healthcare. Biosensors (Basel). (2023) 13:205. doi: 10.3390/bios13020205 36831971 PMC9953568

[5] Natto ZS Alshehri MM Alghamdi FK. Infection control practices at the dental clinics in Jeddah, Saudi Arabia. J Multidiscip Healthc. (2021) 14:2951–7. doi: 10.2147/JMDH.S330567, PMID: 34720586 PMC8550542

[6] Coulthard P. The oral surgery response to coronavirus disease (COVID-19). Keep calm and carry on? Oral Surg. (2020) 13:95–7. doi: 10.1111/ors.12489

[7] Ahmed MA Jouhar R Ahmed N Adnan S Aftab M Zafar MS. Fear and practice modifications among dentists to combat novel coronavirus disease (COVID-19) outbreak. Int J Environ Res Public Health. (2020) 17:2821. doi: 10.3390/ijerph17082821, PMID: 32325888 PMC7216192

[8] Kamate SK Sharma S Thakar S Srivastava D Sengupta K Hadi AJ. Assessing knowledge, attitudes and practices of dental practitioners regarding the COVID-19 pandemic: a multinational study. Dent Med Probl. (2020) 57:11–7. doi: 10.17219/dmp/119743, PMID: 32307930

[9] Doubravská L Htoutou Sedláková M Fišerová K Klementová O Turek R Langová K. Bacterial community- and hospital-acquired pneumonia in patients with critical COVID-19-a prospective monocentric cohort study. Antibiotics (Basel). (2024) 13:192. doi: 10.3390/antibiotics13020192 38391578 PMC10886267

[10] Haischer MH Beilfuss R Hart MR Opielinski L Wrucke D Zirgaitis G. Who is wearing a mask? Gender-, age-, and location-related differences during the COVID-19 pandemic. PLoS One. (2020) 15:e0240785. doi: 10.1371/journal.pone.0240785, PMID: 33057375 PMC7561164

[11] He W Cai D Geng G Klug D. Factors influencing wearing face mask in public during COVID-19 outbreak: a qualitative study. Disaster Med Public Health Prep. (2022) 17:e141. doi: 10.1017/dmp.2022.52 35241205 PMC9002151

[12] Chou YH Lin YC Lee MH Huang YT Liu PF Huang CL. Highly educated patients have lower dental compliance during the COVID-19 pandemic: an observational study. BMC Oral Health. (2022) 22:284. doi: 10.1186/s12903-022-02307-x, PMID: 35820884 PMC9274183

[13] Lertsakulbunlue S Kittisarapong P Pikulkaew S Pusayapaibul P Tangtongsoonthorn A Wichaiboon C. What sustains mask-wearing behavior among elders in a rural community in the post-COVID-19 era: an exploratory mixed-methods study. Behav Sci (Basel). (2023) 13:678. doi: 10.3390/bs13080678 37622818 PMC10451204

[14] Xia T Xu X Ding S. A study of the relationship between social anxiety and mask-wearing intention among college students in the post-COVID-19 era: mediating effects of self-identity, impression management, and avoidance. Front Psychol. (2023) 14:1287115. doi: 10.3389/fpsyg.2023.1287115 38078258 PMC10703186

[15] Cha SE Ku X Choi I. Post COVID-19, still wear a face mask? Self-perceived facial attractiveness reduces mask-wearing intention. Front Psychol. (2023) 14:1084941. doi: 10.3389/fpsyg.2023.1084941, PMID: 36760455 PMC9904203

[16] Beaglehole R Bonita R. Basic epidemiology. Geneva: World Health Organization (1993).

[17] Asadi-Pooya AA Nezafat A Sadeghian S Shahisavandi M Nabavizadeh SA Barzegar Z. Mask wearing hesitancy during the COVID-19 pandemic in South Iran. Disaster Med Public Health Prep. (2022) 16:1789–91. doi: 10.1017/dmp.2021.72, PMID: 33750513 PMC8129672

[18] Zhang W Chen SF Li KK Liu H Shen HC Zhang XC. Mask-wearing behavior during COVID-19 in China and its correlation with e-health literacy. Front Public Health. (2022) 10:930653. doi: 10.3389/fpubh.2022.930653, PMID: 35937248 PMC9354616

[19] Howard MC. Gender, face mask perceptions, and face mask wearing: are men being dangerous during the COVID-19 pandemic? Pers Individ Dif. (2021) 170:110417. doi: 10.1016/j.paid.2020.110417 33052155 PMC7543707

[20] Melzow F Mertens S Todorov H Groneberg DA Paris S Gerber A. Aerosol exposure of staff during dental treatments: a model study. BMC Oral Health. (2022) 22:128. doi: 10.1186/s12903-022-02155-9, PMID: 35428223 PMC9012061

[21] Murakami M. Re-examining the importance of mask-wearing at mass gathering events. Lancet Reg Health Eur. (2022) 18:100423. doi: 10.1016/j.lanepe.2022.100423, PMID: 35655659 PMC9148391

[22] Ko Y Lee SM. The impact of health literacy on consumer knowledge of mask and hand sanitizer use in post-pandemic Korea. Healthcare (Basel). (2025) 13:125. doi: 10.3390/healthcare13020125 39857152 PMC11764667

[23] Ng TKC Fong BYF Law VTS Tavitiyaman P Chiu WK. Mask-wearing intention after the removal of the mandatory mask-wearing requirement in Hong Kong: application of the protection motivation theory and the theory of planned behaviour. Hong Kong Med J. (2025) 31:119–29. doi: 10.12809/hkmj2311274, PMID: 40189797

[24] Kawase A Fukushige M. Expectations regarding the effectiveness of mask-wearing and pandemic fatigue: the experience in Japan. PLoS One. (2025) 20:e0321402. doi: 10.1371/journal.pone.0321402, PMID: 40367129 PMC12077696

[25] Looi KH. Explicating gender disparity in wearing face masks during the COVID-19 pandemic. BMC Public Health. (2022) 22:2273. doi: 10.1186/s12889-022-14630-7, PMID: 36471303 PMC9724360

[26] Parada-Fernández P Herrero-Fernández D Jorge R Comesaña P. Wearing mask hinders emotion recognition, but enhances perception of attractiveness. Pers Individ Dif. (2022) 184:111195. doi: 10.1016/j.paid.2021.111195, PMID: 36540665 PMC9755824

[27] Schmitt DP Long AE McPhearson A O'Brien K Remmert B Shah SH. Personality and gender differences in global perspective. Int J Psychol. (2017) 52:45–56. doi: 10.1002/ijop.12265, PMID: 27000535

[28] Schlegtendal A Eitner L Falkenstein M Hoffmann A Lücke T Sinningen K. To mask or not to mask-evaluation of cognitive performance in children wearing face masks during school lessons (MasKids). Children (Basel). (2022) 9:95. doi: 10.3390/children9010095 35053720 PMC8774884

[29] Zhang K Vilches TN Tariq M Galvani AP Moghadas SM. The impact of mask-wearing and shelter-in-place on COVID-19 outbreaks in the United States. Int J Infect Dis. (2020) 101:334–41. doi: 10.1016/j.ijid.2020.10.002, PMID: 33039614 PMC7544634

[30] Kwan RYC Lee PH Cheung DSK Lam SC. Face mask wearing behaviors, depressive symptoms, and health beliefs among older people during the COVID-19 pandemic. Front Med (Lausanne). (2021) 8:590936. doi: 10.3389/fmed.2021.590936, PMID: 33614680 PMC7892765

[31] Hu R Liu J Xie Y Jiao J Fang Z Lin B. Effects of mask wearing duration and relative humidity on thermal perception in the summer outdoor built environment. Build Simul. (2022) 16:1601–1616. doi: 10.1007/s12273-022-0978-9 PMC9798370 36593872

[32] Schachter EN Lach E Lee M. The protective effect of a cold weather mask on exercised-induced asthma. Ann Allergy. (1981) 46:12–6. PMID: 7458007

[33] Xiao Q Ma Z Li S Liu Y. The impact of winter heating on air pollution in China. PLoS One. (2015) 10:e0117311. doi: 10.1371/journal.pone.0117311, PMID: 25629878 PMC4309400

[34] Li Y Yu J Wang Y Yi J Guo L Wang Q. Cocirculation and coinfection of multiple respiratory viruses during autumn and winter seasons of 2023 in Beijing, China: a retrospective study. J Med Virol. (2024) 96:e29602. doi: 10.1002/jmv.29602, PMID: 38597349

[35] Fu H Chen J. Formation, features and controlling strategies of severe haze-fog pollutions in China. Sci Total Environ. (2017) 578:121–38. doi: 10.1016/j.scitotenv.2016.10.201, PMID: 27836344

[36] Cai DP He YM. Daily lifestyles in the fog and haze weather. J Thorac Dis. (2016) 8:E75–7. doi: 10.3978/j.issn.2072-1439.2016.01.35 26904256 PMC4740148

[37] Jiang XQ Mei XD Feng D. Air pollution and chronic airway diseases: what should people know and do? J Thorac Dis. (2016) 8:E31–40. doi: 10.3978/j.issn.2072-1439.2015.11.50, PMID: 26904251 PMC4740163

